# A pilot study on staged surgery by delta video-assisted anal fistula treatment (dVAAFT) for complex anal fistula

**DOI:** 10.1007/s13304-023-01607-3

**Published:** 2023-08-03

**Authors:** Marco La Torre, Giorgio La Greca, Enrico Fiori, Vito D’Andrea, Simone Maria Tierno, Federica Tommasini, Ugo Grossi, Gaetano Gallo

**Affiliations:** 1 Department of Surgery, Sapienza University of Rome, Rome, Italy; 2 Unit of General Surgery, Ospedale ‘Umberto I’, Enna, Italy; 3 Department of Surgery, Ospedale Figlie di San Camillo Vannini, Rome, Italy; 4 Department of Surgery, Ospedale Grassi di Ostia, Rome, Italy; 5 Department of Surgery, Oncology and Gastroenterology – DISCOG, University of Padua, Padua, Italy; 6 Surgery Unit 2, Regional Hospital Treviso, AULSS2 Marca Trevigiana, Treviso, Italy

**Keywords:** VAAFT, Complex anal fistula, Seton placement

## Abstract

Complex anal fistulas (CAF) present a challenge in achieving healing while preserving anal sphincter function. This study aims to introduce a novel staged approach for CAF treatment, combining video-assisted anal fistula treatment (VAAFT), seton placement, and other staged approaches. Consecutive patients with CAF underwent the staged approach involving VAAFT and seton placement. Data on patient demographics, fistula characteristics, and operative findings were collected. Pre-operative work-up included clinical evaluation, endoanal ultrasonography (EAUS), and magnetic resonance imaging (MRI). Surgical techniques and outcomes were evaluated. Eighteen patients (median age 38 years) were included. Misplacement of a previously placed seton was observed in 64% of cases. VAAFT combined with seton placement achieved simplification and healing of secondary tracts in 66% of cases. Operative times significantly decreased across interventions. At a median follow-up of 14 months, complete healing was achieved in 2 patients, with 1 patient demonstrating persistence of the fistula. Post-operative complications were observed in 11% of patients, with no deterioration in continence. The staged approach combining VAAFT, seton placement, and staged procedures offers a potential solution for treating CAF. VAAFT provides diagnostic and therapeutic benefits, simplifying the fistula anatomy and optimizing seton placement. The approach allows subsequent procedures based on individual fistula characteristics.

**Supplementary Information:**

The online version contains supplementary material available at 10.1007/s13304-023-01607-3.

## Introduction

Complex anal fistulas (CAF) pose a significant challenge for both patients and surgeons, accounting for approximately 30% of all anorectal fistulas [1, 2]. Managing CAF requires a delicate balance between achieving fistula healing and preserving the integrity of the anal sphincters [3].

In recent decades, various sphincter-sparing techniques have been introduced to reduce the risk of post-operative incontinence following radical surgery. These techniques include advancement flap repair, fibrin glue injection, fistula plug insertion, ligation of the inter-sphincteric tract (LIFT), and fistula laser closure (FiLaC) [4]. In 2011, Meinero and Mori introduced the video-assisted anal fistula treatment (VAAFT) technique as a promising approach for CAF.[5] VAAFT involves the use of an endoscopic fistuloscope during the preoperative phase, allowing direct visualization of primary and secondary tracts, as well as the identification of deep abscess cavities [6]. Sphincter preservation is achieved through diathermy coagulation of the inflammatory tissue, without disruption or damage to the anal sphincter complex [7].

Staged surgery, incorporating partial fistulotomy/fistulectomy, the placement of a loose seton, and other procedures, has also emerged as an alternative strategy to promote CAF healing. The primary goal of staged surgery is to shorten complex fistula tracts and simplify the overall fistula anatomy.

The objective of this study is to determine the safety and feasibility of a novel staged approach for the treatment of CAF, combining VAAFT, fistula drainage with a loose seton, and staged fistulectomy/fistulotomy. By utilizing this approach, we aim to enhance the likelihood of successful fistula treatment while preserving sphincter function. Furthermore, evaluating operative outcomes and medium-term results will provide valuable insights into the effectiveness and safety of this staged approach. This study contributes to the ongoing efforts to optimize the management of CAF, ultimately improving patient outcomes and quality of life.

## Methods

Consecutive patients with CAF who underwent a staged approach involving VAAFT and seton placement were enrolled in this study between January 2019 and December 2022 at a tertiary colorectal center. The study was reported in accordance with the STROBE checklist (appendix 1).

Patient data were collected and analysed from our prospectively maintained database. Information on patient demographics, fistula type and location, prior attempts at repair, and operative findings were recorded.

The inclusion criteria comprised patients with CAF, including anterior fistulas in females, high transphincteric fistulas, suprasphincteric fistulas, fistulas with ramifications, horseshoe fistulas, and those with multiple tracts. Patients under the age of 18 and those with inflammatory bowel disease were excluded from the study.

All patients underwent a comprehensive pre-operative work-up assessment, including clinical evaluation, endoanal ultrasonography (EAUS), and magnetic resonance imaging (MRI). EAUS was performed with a 6–16 MHz radial transducer (type 2052) in the left-lateral position using a B-K Medical (Herlev, Denmark) endoprobe. MRI scans included axial, coronal, and oblique planes, with the sagittal fast spin-echo T2-weighted sequence initially employed to visualize the entire pelvis and anal canal and obtain proper orientation. Fistulas were classified based on clock position, with anterior fistulas having an external opening between 10 and 2 o’clock, posterior fistulas between 4 and 8 o’clock, and lateral fistulas between 2 and 4 and 8 and 10 o’clock.

All elective surgical procedures were consistently performed by the same experienced colorectal surgeon (MLT), who received training from the inventor of the VAAFT technique.

### Surgical technique and post-operative steps

The VAAFT procedure was performed following the standard technique described elsewhere [7]. However, in contrast to the standard technique, closure of the internal orifice was not performed in our approach. Instead, all patients underwent seton placement to facilitate drainage of deep cavities and secondary tracts. Additionally, a partial fistulotomy was performed, limited to the external anal sphincter plane, to shorten the tract(s) and simplify the fistula anatomy. Patients were followed up for a period of three months after the initial surgery, thence a pelvic MRI was conducted to assess the radiological healing of deep cavities and secondary tracts following VAAFT. If the MRI demonstrated successful simplification of the fistula, the patient proceeded to the second stage of the procedure, aimed at closing the main fistula tract. Fistulotomy was the preferred technique for low fistula tracts, while FiLaC and LIFT were chosen for residual high trans-sphincteric tracts.

In cases where simplification of the fistula could not be achieved based on the MRI findings, a repeat VAAFT procedure with a new drainage technique was performed, and seton placement was re-implemented after the redo surgery.

Patients who achieved radiological healing of deep cavities and secondary tracts on pelvic MRI at the three-month follow-up were referred for the final surgical procedure.

Anal continence was assessed preoperatively and three months postoperatively using the Vaizey incontinence score, which ranges from 0 (indicating perfect continence) to 24 (indicating total incontinence) [8]. Postoperative complications were classified according to the Clavien-Dindo classification system [9].

Persistence of the disease and failure of the technique was defined as incomplete healing of the external orifice(s), as well as the persistence of discharge from internal/external orifice(s) during the first six months. Recurrences were defined as new radiologically and/or clinically confirmed onset of the fistula after primary healing.

### Statistical analysis

Number and percentages were used for the categorical variables’ description, whereas medians and interquartile ranges (IQR) were used for the continuous variables’ description. Friedman test was used to compare operative times across the first, second and third interventions. Pearson Chi-squared test or Fisher exact test was used to compare categorical variables as appropriate.

Binary logistic regression models were used to assess the correlation between fistula type (i.e., transphincteric vs. supra/extrahincteric), the location of the internal orifice (i.e., anterior vs. posterior), and the radiological improvement following the initial treatment attempt or clinical healing at the one-year postoperative mark.

All statistical analyses were conducted using SPSS ver. 29.0 (IBM, Armonk, NY, USA), and a *p*-value of less than 0.05 was considered statistically significant.

## Results

A total of 18 patients (5 female; median age [IQR], 38 [35–49]) were included in the study (Table 1). Among them, 12 (66%) patients achieved simplification of the CAF with healing of the secondary tracts following the VAAFT procedure combined with loose seton placement. Fistula type (*p* = 0.122) and location of the internal orifice (0.400) did not correlate with MRI improvement 3 months after the first treatment attempt.

**Table 1:** Patients’ characteristics

Age in years, median (IQR)	38 (35–49)
Gender, males (%)	13 (72)
Recurrent fistula, *n* (%)	14 (78)
Number of external orifices, median (IQR)	1 (1–2)
Internal orifice location, *n* (%) Anterior Posterior	4 (22) 14 (78)
External orifice location, *n* (%) Anterior Posterior Left lateral Right lateral	4 (22) 8 (45) 2 (11) 4 (22)
Type of fistula–Park’s classification, *n* (%) Trans-sphincteric Extra-sphincteric Supra-sphincteric	13 (72) 3 (17) 2 (11)
Location of the secondary tract, *n* (%) Ischio-rectal Pelvi-rectal Horseshoe	9 (50) 7 (39) 2 (11)
Number of previous surgical treatments, mean (range)	1.7 (0–8)

**Fig. 1:**
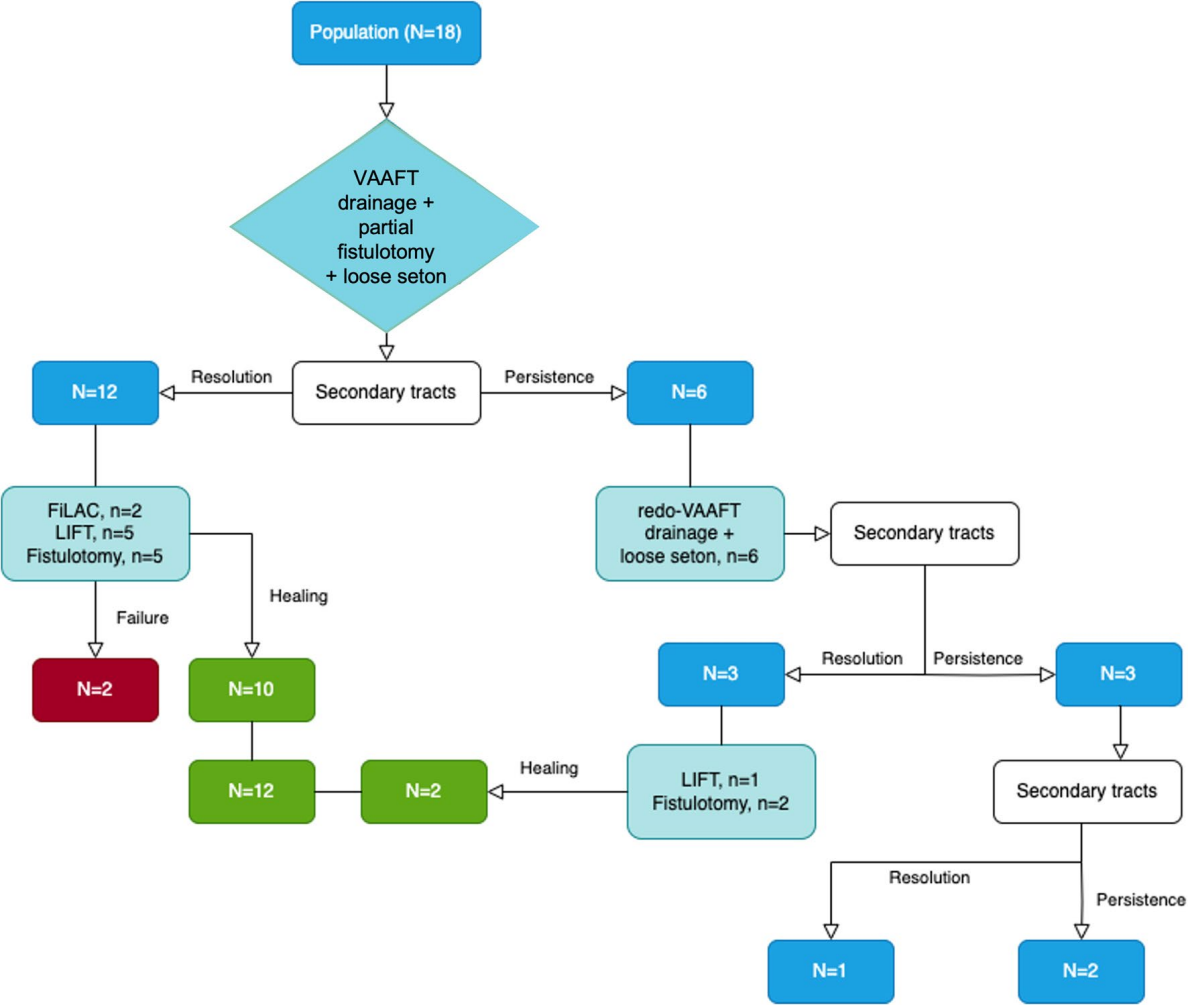
Study population

Misplacement of the previously placed seton was observed in 9 out of 14 cases (64%). All patients in this subgroup underwent a second-stage procedure. Specifically, 5 patients underwent fistulotomy and primary sphincteroplasty, 5 patients underwent LIFT, and 2 patients underwent the FiLaC procedure (Fig. 1).

Six patients (33%) presented persistence of secondary tracts at the 3-month MRI following the first stage VAAFT procedure with seton placement. In these cases, a repeat VAAFT procedure with seton drainage was performed, and a new MRI evaluation was obtained after an additional 3 months (Fig. 1).

Among these 6 patients, 3 achieved closure of the secondary tracts and abscesses after the repeat VAAFT procedure combined with seton placement, and they were subsequently referred for the final treatment of the fistula. Specifically, 2 patients underwent fistulotomy and sphincteroplasty, and 1 patient underwent LIFT.

Operative times significantly decreased from the first to the third interventions (median [IQR] 1st intervention, 60 [48–75] minutes vs. 2nd intervention, 48 [44–50] minutes vs. 3rd intervention, 40 [30–45] minutes; *p* = 0.013).

After a median follow-up of 14 (12–17) months, complete healing was achieved in 2 patients, while 1 patient still demonstrated persistence of the fistula (Table 2 and Fig. 1). Fistula type (*p* = 0.439) and location of the internal orifice (0.734) did not correlate with clinical healing at 12 months post-operatively.

**Table 2:** Surgical outcomes

Surgical outcomes	
Success after 12 months of follow-up	12 (66%)
Failure of staged treatment	6 (34%)
Patients with persistence of complex fistula tracts	2 (11%)

The post-operative complication rate was 11% (2 out of 18 patients), with two Clavien-Dindo grade I complications observed (1 case of postoperative bleeding and 1 case of urinary retention). None of these patients required re-operation. No deterioration in faecal continence was observed between the pre- and post-operative Vaizey scores.

## Discussion

None of the currently available sphincter-saving techniques have demonstrated unequivocal success in treating high CAF [10]. Ligation of the intersphincteric fistula tract (LIFT) has been deemed ineffective and even contraindicated for patients with CAF involving secondary deep tracts [7]. Fistula laser closure (FiLAC) has shown improved outcomes when preceded by seton placement to reduce the recurrence and persistence of the disease [11].

Some authors have proposed the use of seton placement combined with staged surgery for CAF, aiming to drain associated abscess cavities, reduce inflammatory tissue, promote healing of deep secondary tracts, and decrease the overall length of the fistula [12].

Recently, Iqbal and Tozer [13] emphasized the non-curative properties of VAAFT, which focuses on direct visualization and debridement of the fistula tract or specific areas of fistula morphology. This approach has resulted in symptomatic improvement for patients (palliative intent) and downstaging of CAF (staged or delta VAAFT) [14].

The blind insertion of the probe carries the risk of misidentifying the main tract and potentially missing secondary fistula branches, leading to a higher risk of seton misplacement and fistula recurrence. In our series, seton misplacement was observed in 9 out of 14 patients (64%), confirming the high risk associated with blind probe insertion. In contrast, the replacement of the seton under the guidance of the fistuloscope during dVAAFT resulted in no instances of misplacement, ensuring accurate drainage of all tracts. Indeed, a recent systematic review reported a high internal opening detection rate of 97.6% with VAAFT [15].

In this context, VAAFT can be regarded as a novel and effective first-stage procedure, enabling the identification and drainage of deep fistula tracts by providing clarity on the fistula anatomy and avoiding residual inflammatory tissue along with optimization of seton placement. Therefore, the procedure aims to simplify the fistula prior to secondary-stage procedures. Furthermore, the ability to use endobrush and unipolar electrodes under direct vision reduces surgical scarring and minimizes damage to the sphincter complex, preserving its function and improving patient satisfaction.

Moreover, staged surgery involving VAAFT and seton placement does not limit future surgical approaches or strategies during subsequent stages. Indeed, in our study, patients underwent second-stage procedures such as fistulotomy, LIFT, and FiLAC, all of which yielded effective outcomes. Furthermore, the gradual decrease in operative time indicates a progressive reduction in the complexity of the interventions.

In their systematic review, Emile et al. [16] underscored the diagnostic role of VAAFT, demonstrating that recurrence rates may partly depend on the method of closure of the internal opening. The significant overall recurrence rate of 29% at 1 year and 63% at 5 years observed after VAAFT in our experience [17] may partially reflect a relatively complex patients’ population, with 25% having a suprasphincteric or extrashpincteric fistula.

In our study, the combination of VAAFT and loose seton placement in the first-stage procedure, along with partial fistulotomy up to the external anal sphincter, yielded successful outcomes in 66% of cases.

It is important to acknowledge the limitations of our study, primarily its retrospective nature and small sample size. Caution should be taken when interpreting our results, as they reflect a specific population with CAF. To the best of our knowledge, this is the first study to focus on the diagnostic and non-curative role of VAAFT.

In summary, VAAFT represents a promising, reasonable, and viable option for patients with CAF who have limited surgical alternatives. Thorough and comprehensive informed consent is crucial to adequately inform patients about the complexity of the fistula and the possibility of repeated procedures.

Furthermore, the long-term follow-up of patients who undergo VAAFT and staged surgery is essential to assess the durability of outcomes and the potential for late recurrence. Future studies with larger sample sizes and longer observation periods are needed to validate the findings of this study and further evaluate the effectiveness and safety of VAAFT as a treatment option for CAF. Additionally, comparative studies comparing VAAFT with other surgical techniques can provide valuable insights into the optimal management approach for these challenging cases.

### Supplementary Information

Below is the link to the electronic supplementary material.

Supplementary file1 (PDF 392 kb)

## Data Availability

The data that support the findings of this study are available from the corresponding authors [UG and GG] upon reasonable request.

## References

[1] Sandborn WJ (2003). AGA technical review on perianal Crohn’s disease. Gastroenterology.

[2] Garg P, Sodhi SS, Garg N (2020). Management of complex cryptoglandular anal fistula: challenges and solutions. Clin Exp Gastroenterol.

[3] Pucciani F (2018). Post-surgical fecal incontinence. Updates Surg.

[4] Steele SR (2011). Practice parameters for the management of perianal abscess and fistula-in-ano. Dis Colon Rectum.

[5] Meinero P, Mori L (2011). Video-assisted anal fistula treatment (VAAFT): a novel sphincter-saving procedure for treating complex anal fistulas. Tech Coloproctol.

[6] Walega P, Romaniszyn M, Nowak W (2014). VAAFT: a new minimally invasive method in the diagnostics and treatment of anal fistulas–initial results. Pol Przegl Chir.

[7] La Torre M (2020). Lift and VAAFT for high trans-sphincteric anal fistula: a single center retrospective analysis. Int J Colorectal Dis.

[8] Vaizey CJ (1999). Prospective comparison of faecal incontinence grading systems. Gut.

[9] Dindo D, Demartines N, Clavien PA (2004). Classification of surgical complications: a new proposal with evaluation in a cohort of 6336 patients and results of a survey. Ann Surg.

[10] Elshamy MT (2022). A pilot randomized controlled trial on ligation of intersphincteric fistula tract (LIFT) versus modified parks technique and two-stage seton in treatment of complex anal fistula. Updates Surg.

[11] Adegbola SO (2021). Emerging data on fistula laser closure (FiLaC) for the treatment of perianal fistulas; patient selection and outcomes. Clin Exp Gastroenterol.

[12] Tozer P (2019). Video-assisted anal fistula treatment (VAAFT) assisted seton placement - a video vignette. Colorectal Dis.

[13] Iqbal N, Tozer P (2022). How does VAAFT fit into the world of clinical and academic anal fistula?. Tech Coloproctol.

[14] Chase TJG (2021). VAAFT for complex anal fistula: a useful tool, however, cure is unlikely. Tech Coloproctol.

[15] Tian Z (2022). Video-assisted anal fistula treatment for complex anorectal fistulas in adults: a systematic review and meta-analysis. Tech Coloproctol.

[16] Emile SH (2018). A Systematic review and meta-analysis of the efficacy and safety of video-assisted anal fistula treatment (VAAFT). Surg Endosc.

[17] La Torre MG, Micarelli MA, Fiori E, D’Andrea V, Grossi U, Tierno SM, Tomassini F, Gallo G, Long term results of Video-assisted Anal Fistula Treatment (VAAFT) for complex anal fistula: another shattered dream? Colorectal Dis., 2023. *Submitted*10.1111/codi.16732 37658596

